# Neural activity during inhibitory control predicts suicidal ideation with machine learning

**DOI:** 10.1038/s44277-024-00012-x

**Published:** 2024-07-08

**Authors:** Jason Nan, Gillian Grennan, Soumya Ravichandran, Dhakshin Ramanathan, Jyoti Mishra

**Affiliations:** 1 Neural Engineering and Translation Labs, University of California, San Diego, La Jolla, CA USA; 2 Department of Bioengineering, University of California, San Diego, La Jolla, CA USA; 3 Department of Psychiatry, University of California, San Diego, La Jolla, CA USA; 4 Department of Mental Health, VA San Diego Medical Center, San Diego, CA USA; 5 Center of Excellence for Stress and Mental Health, VA San Diego Medical Center, San Diego, CA USA

**Keywords:** Predictive markers, Psychiatric disorders

## Abstract

Suicide is a leading cause of death in the US and worldwide. Current strategies for preventing suicide are often focused on the identification and treatment of risk factors, especially suicidal ideation (SI). Hence, developing data-driven biomarkers of SI may be key for suicide prevention and intervention. Prior attempts at biomarker-based prediction models for SI have primarily used expensive neuroimaging technologies, yet clinically scalable and affordable biomarkers remain elusive. Here, we investigated the classification of SI using machine learning (ML) on a dataset of 76 subjects with and without SI(+/−) (*n* = 38 each), who completed a neuro-cognitive assessment session synchronized with electroencephalography (EEG). SI+/− groups were matched for age, sex, and mental health symptoms of depression and anxiety. EEG was recorded at rest and while subjects engaged in four cognitive tasks of inhibitory control, interference processing, working memory, and emotion bias. We parsed EEG signals in physiologically relevant theta (4-8 Hz), alpha (8–13 Hz), and beta (13–30 Hz) frequencies and performed cortical source imaging on the neural signals. These data served as SI predictors in ML models. The best ML model was obtained for beta band power during the inhibitory control (IC) task, demonstrating high sensitivity (89%), specificity (98%). Shapley explainer plots further showed top neural predictors as feedback-related power in the visual and posterior default mode networks and response-related power in the ventral attention, fronto-parietal, and sensory-motor networks. We further tested the external validity of the model in an independent clinically depressed sample (*n* = 35, 12 SI+) that engaged in an adaptive test version of the IC task, demonstrating 50% sensitivity and 61% specificity in this sample. Overall, the study suggests a promising, scalable EEG-based biomarker approach to predict SI that may serve as a target for risk identification and intervention.

## Lay Summary

This study achieves a high-accuracy machine learning model that can classify an individual as having suicidal ideation or not from source-localized EEG signals captured during an inhibitory control task. In addition, we have identified key brain regions that drive this model.

## Introduction

In the last twenty years, suicide rates have increased by almost 41% and now constitute the tenth leading cause of death in the United States [1]. Several well-studied social and psychological factors have been shown to contribute to the complex etiology of suicidality including social isolation, psychological distress, chronic conditions, financial difficulties, and low esteem [2, 3]. With the advent of the COVID-19 pandemic, the prevalence of suicide risk factors like mental health illnesses has starkly increased, perhaps contributing to the doubled incidence rate of suicidal ideation (SI) [4].

Machine learning (ML) models may be a promising approach for the early identification of individuals at risk for suicide or identifying additional targets for treatment. Previous studies have utilized ML models to aggregate social media data as a construct for suicidal risk identification and early detection of mental health concerns, like depression [5–7]. Models using self-report data about lifestyle factors, such as early life temperament and parental mental health, have also proven to contribute to an understanding of predictors of suicide [8, 9], though these models show overall relatively low accuracy. In addition to these psychological and social factors predicting suicide, efforts have been made to identify biological predictors [10–13].

Relevant to this study, neural changes associated with suicide risk have been explored using magnetic resonance imaging (MRI/functional MRI) [14]. This study suggested that a history of SI was linked with alterations/ reduced activity or connectivity of ventral and dorsolateral prefrontal cortical areas. ML approaches have also identified alterations in default mode network and sensorimotor cortical areas in association with SI [15, 16]. There are several challenges with the large-scale adoption of these methods for screening or biological targeting. First, much of this prior literature did not properly control for age/gender or (more importantly) depression history. Thus, differences observed may not be directly linked with SI per se, but instead may be a consequence of other uncontrolled differences between groups [17, 18]. Second and more importantly, the initial upfront expense, as well as the per-session cost of MRIs, makes this technology not always immediately accessible, complicating efforts to use this as a screening tool to identify those at elevated risk of SI. Thus, efforts at identifying biomarkers from electroencephalography (EEG) may have much potential scalable utility.

For depression, recent work has shown that EEG can be useful in understanding and predicting treatment [19]. A review noted that models utilizing multi-modal data types consistently showed higher performance compared to singular data types in predicting therapeutic outcomes in depression [20]. Beyond simple predictions of depression using neuroimaging [21], other studies have attempted to use ML to identify differences in specific regions of the brain that are associated with depression [22, 23]. A recent study also used ML to predict the efficacy of pharmacological treatments for depression based on established pharmacogenomic biomarkers [24].

There have been fewer efforts at understanding EEG biomarkers of suicidality – and results have been generally mixed. One study examining differences in EEG after suicide attempt compared to healthy controls found no differences in alpha-band connectivity [25]. Another group identified reduced theta-band activity in individuals with SI compared to those without [26] though notably, in this study excluded individuals there was a difference in depression and anxiety symptom severity between groups. Another group found elevated frontal gamma power in individuals with SI compared to those without [27], though this study did not report or control for depression severity. Studies that have examined other biomarkers, such as alpha-asymmetry, have likewise found limited relationships with SI [28]. Finally, an interesting study showed differences in EEG micro-states between individuals with depression and SI compared to healthy controls [29] – but again, the presence of depression only in the SI group presents a confound.

Thus, to the best of our knowledge, there have been no studies with EEG that have leveraged ML-based classification approaches for SI and that have included age/sex/depression/anxiety-matched controls. In addition, most ML approaches to date across both fMRI and EEG, have leveraged resting-state data. While resting state data may reveal basic biological differences in individuals with SI, it does not provide any insight into differential neural processing that may occur during cognitive tasks. Neurocognitive deficits are a known potential risk factor for SI [30]. Various cognitive tasks have been linked to suicidality, for example, suicidal individuals exhibit impaired inhibitory control and emotional dysregulation [31, 32]. Studies have also shown that individuals with SI display impacted interference processing efficiency [33]; poor interference processing may predispose individuals to a sustained state of hypervigilance and inefficient allocation of cognitive load to sensory stimuli [34, 35]. Deficits in working memory have also been associated with suicidality as a mediating factor of increased negative affect. [36, 37]. Furthermore, previous studies have widely observed a negative bias in emotional processing as a key vulnerability factor for behavioral impulsivity associated with diagnoses such as major depressive disorder, ADHD, and SI [38, 39].

We hypothesized that an analysis of EEG signals gathered during relevant cognitive tasks would provide an improved ability to identify biomarkers/classification of individuals with SI. To address shortcomings of previous research, we employed a pair study design with a sample size of 76 individuals controlling for age, sex, and depression/anxiety symptoms. Given the literature proposing deficits in cognitive control in individuals with SI, our study focused on analyzing EEG spectral signals during cognitive tasks aimed at assessing inhibitory control, interference processing, working memory, and emotion bias, alongside resting state data, and we analyzed power across common frequency bands of interest (theta, alpha, and beta).

## Materials and Methods

### Main study participants

A total of 76 human subjects participated in the study (median ± median absolute deviation (MAD) age: 23 ± 7.6 years, range: 18–72 years, 33 males). All subjects were fluent in English. Each participant gave written informed consent in accordance with the Declaration of Helsinki before participating in the experiment. All experimental procedures were approved by the Institutional Review Board of the University of California San Diego (UCSD) (protocol #180140). Participants were recruited from the San Diego community using the Research Match registry for potential research volunteers. Data collection took place from Spring 2018 to Spring 2020.

#### Groups matched for demographics and mental health characteristics

The 76 study subjects were evenly divided into two groups with and without suicidal ideation (SI+/−), see Table 1. These subjects were part of a larger dataset of 324 participants from the San Diego community representative sample. For inclusion in this particular study, participants must have self-reported demographics and mental health, and undergone the neuro-cognitive testing procedures. Subjects did not undergo a structured clinical interview to determine inclusion/exclusion. Participants self-reported any clinical diagnoses or medications. No participants reported unstable or serious medical illness, neurological disorder history of a seizure disorder, any psychotic disorder or current active psychotic symptoms, or active substance abuse/dependence that would have been cause for exclusion. In the larger dataset of 324 subjects, there were 38 SI+ subjects that were included in this study, and corresponding to these, we chose 38 SI- individuals with similar age/sex/anxiety/depression characteristics in order to obtain balanced groups. Depression symptoms were self-reported on the Patient Health Questionnaire 9-item scale, PHQ9[40], and anxiety was self-reported on the Generalized Anxiety Disorder 7-item scale, GAD7 [41]. Ethnicity and socio-economic status as measured by the Family Affluence Scale[42] also did not significantly differ for participants. All matching was assessed by significance testing between the two groups and ensuring *p* > 0.05 for all matched variables. Subjects differed in ratings of suicidal ideation (SI+/−) based on responses on the Columbia Suicide Severity Rating Scale (C-SSRS [43]); SI- individuals had C-SSRS scores of 0, and SI+ individuals had scores of 1-3 (key: 1- Wishes to be dead; 2-Non-specific active suicidal thoughts; and 3-Active Suicidal Ideation and Any Methods (Not Plan) without intent to act). No suicidal attempts were reported. The SI+ group had 7 subjects and the SI- group had 5 subjects with prescribed antidepressant medications, respectively.

**Table 1:** Demographic and mental health characteristics for main participants.

Demographics	SI +	SI-	*p*-value
	Median ± mad	Median ± mad	
Age	23 ± 7.5	22.5±7.6	0.64
Sex *n* (%)			0.49
Male	18 (47.4)	15 (39.5)	
Female	20 (52.6)	23 (60.5)	
Ethnicity *n* (%)			0.07
White	12 (31.6)	24 (63.2)	
Black/African American	2 (5.3)	0 (0)	
Asian	15 (39.5)	6 (15.8)	
Native American	2 (5.3)	0 (0)	
More than one race	6 (15.8)	4 (10.5)	
Other	1 (2.6)	4 (10.5)	
SES	4.0 ± 1.5	5 ± 1.53	0.15
Anxiety (GAD7)	8.5 ± 4.1	9.0 ± 3.9	0.78
Depression (PHQ9)	12 ± 3.3	11 ± 2.4	0.35

Data are shown for individuals with suicidal ideation (SI+, *n* = 38) vs. no suicidal ideation (SI-, *n* = 38). Variables were compared between groups using the Wilcoxon rank sum test, except categorical variables of sex and ethnicity were compared between groups using *χ*^*2*^ (Chi-Square) statistics. *SES* socio-economic score, *GAD7* generalized anxiety disorder 7-item scale, *PHQ9* patient health questionnaire 9-tem scale, *mad* median absolute deviation.

### External validation study participants

Additionally, a total of 35 human subjects recruited from two depression clinics participated in the study as a blind validation testing set (median ± MAD age: 57 ± 10 years, range: 19-74 years, 16 males, 12 SI+). All subjects were fluent in English. Each participant gave written informed consent in accordance with the Declaration of Helsinki before participating in the experiment. All experimental procedures were approved by the Institutional Review Board of the University of California San Diego (UCSD) (protocol #180140) and the Veterans Affairs San Diego Health Systems (protocol #H200041). Data collection took place during Spring 2022–Fall 2023. For inclusion/exclusion, this clinical sample underwent the Structured Clinical Interview for DSM-5 (SCID) and was confirmed to have clinical depression, and without any unstable or serious medical illness, neurological disorder or history of a seizure disorder, any psychotic disorder, or current active psychotic symptoms, or active substance abuse/dependence. In this sample, 9/12 SI+ and 18/23 SI- subjects were on prescribed antidepressant medications.

### Neuro-cognitive assessments

**Fig. 1:**
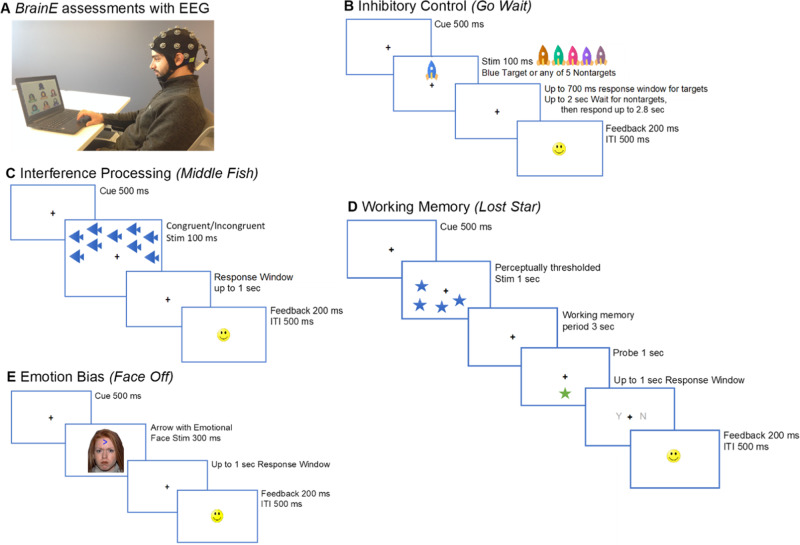
Schematic layout and stimulus sequence of neuro-cognitive tasks. All participants performed four neuro-cognitive assessment tasks implemented on the *BrainE* platform in a single experimental session. **A** Snapshot of the computerized cognitive assessment with EEG; **B** Inhibitory Control (IC) task; **C** Interference Processing (IP) task; **D** Working Memory (WM) task; **E** Emotion Bias (EB) task. Besides these tasks, eyes-closed resting state data was also acquired for 3 minutes. ITI Inter-trial interval. The individual whose face appears in **A** gave signed consent for their likeness to be published in this article.

Standard neuro-cognitive assessments were deployed on the Unity game engine on the *BrainE* (short for Brain Engagement) platform [44] that we have now used in several studies [34, 35, 45–54]. EEG data were acquired simultaneously for all cognitive tasks at 250 Hz sampling frequency at 24-bit resolution. EEG acquisition used a 24-channel Smarting^TM^ wireless EEG amplifier with saline-soaked electrodes in a 10–20 standard layout for rapid recordings scalable to any clinical setting [45]. The Lab Streaming Layer [55] protocol was used to timestamp each stimulus/response event in each cognitive task and synchronize timestamps with the EEG recordings. Study participants engaged with *BrainE* neuro-cognitive assessments on a Windows-10 laptop sitting at a comfortable viewing distance. Participants underwent four cognitive assessment modules - inhibitory control (IC), interference processing (IP), working memory (WM), emotion bias (EB), as well as rest, with all recordings completed within a 40 minute (min) session [45]. Figure 1 shows the stimulus sequence in each task. Full descriptions of each task can be found in the **Supplementary Materials and Methods**.

### Data processing

Behavior and neural data analyses were conducted using a uniform processing pipeline published in several of our studies [47–49, 52, 56, 57], and detailed in **Supplementary Materials and Methods**.

Behavioral metrics included d’ signal detection sensitivity, response time (RT), and consistency of response.

For EEG data, some participants had corrupted/missing EEG data: two subjects had missing data for the IC task, and three subjects were missing IP, WM, and EB task data. The structure of all four cognitive tasks shown in Fig. 1 was identical, consisting of sequential stages: fixation cue, stimulus onset (stim), response (resp), and feedback (fdbk). Hence, for all four tasks, we analyzed these 4 distinct time periods to understand which physiological period is most important for predictive modeling. Cue period activity was averaged in the 500 msec period post-cue, stimulus peak processing activity was averaged in the 100-500 msec period post-stimulus onset; response (resp) activity was averaged 50-150 msec post-response and feedback (fdbk) activity was averaged 100-400 msec post-feedback. These time periods were constrained by their event durations during each task trial and our prior identification of peak stimulus processing periods [45]. Also, all clean task trials were used for analysis of the cue and stimulus onset events since they precede decision-making. Only accurate trials were considered for the response and feedback events due to the sparsity of incorrect trials across all tasks and subjects (mean ± standard deviation (std) of the percentage of incorrect trials across tasks and subjects: 9.36 ± 10.38%). For resting state data, the entirety of the pseudo-random epoched data was averaged in time.

**Fig. 2:**
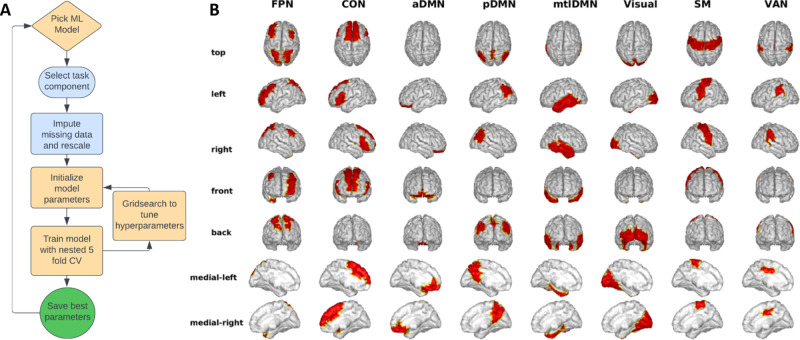
Modeling process and brain networks modeled. **A** Model training pipeline. Starting at the top, a machine learning (ML) model was chosen as either a Logistic Regression (LR) Decision Tree (DT), or Multilayer Perceptron (MLP), applied to each tested dataset described in the Methods. We imputed any missing data and rescaled each variable as basic preprocessing steps. Then we initialized the model parameters and trained the ML model with a 5-fold stratified cross-validation (CV) approach. We looped through this process until all model parameters had been tested, a process called hyper-parameter tuning using grid search. The best parameter set and scores were saved for each combination of dataset and model; the resulting scores are shown in Fig. 3. **B** Data on each cognitive task was modeled as power in eight source localized brain networks as follows: Fronto- Parietal Network (FPN), Cingulo-Opercular network (CON), anterior Default Mode Network (aDMN), posterior Default Mode Network (pDMN), medial temporal lobe Default Mode Network (mtlDMN), Visual network (Visual), Sensory Motor network (SM), and the Ventral Attention Network (VAN).

In EEG processing, we analyzed both scalp channel and cortical source localized data in our models to investigate which may have greater predictive power to classify SI+/− individuals. For this, EEG scalp data were organized into 7 electrode groups: frontal medial, frontal left, frontal right, central, posterior occipital medial, posterior occipital left, and posterior occipital right. Regions of Interest (ROIs) in source space were also grouped into 8 canonical brain networks shown in Fig. 2B. Electrodes and ROIs in each grouping/network are also available in Tables S1 and S2.

### A priori defined ML model variables and datasets

Since we only had *N* = 76 total main subjects, we chose subsets of processed variables in our SI+/− classification models, defined a priori, and constrained by cognitive task and biology as described below. Since demographics and mental health were equivalent between SI+/− groups (Table 1), these variables were not modeled. Our primary hypothesis was that neural data in cortical source space brain networks within specific cognitive tasks may show the greatest separation between SI+/− subjects. Thus, our primary category of ML classification models was based on task-related source network power. In addition to our primary category, we explored three alternative categories of datasets for modeling to help confirm our primary hypothesis.

### Primary category of ML models

#### Task-related EEG source network power

Each of these datasets corresponded to cortical brain network power on a specific cognitive task (including rest). We opted to use source space-derived data due to the greater spatial resolution of brain regions as well as reducing the effect of volume conduction as can occur in scalp electrodes [58–60]. There were a total of 15 datasets: 4 cognitive tasks plus rest, repeated for each frequency band (theta, alpha, beta). For the 4 cognitive tasks, the variables included the 4 different cognitive events (cue, stim, resp, fdbk) for each of the 8 brain networks so there were a total of 32 feature variables per model. The resting state dataset included just 8 variables associated with the 8 brain networks modeled separately for each frequency band. Data from each frequency band was modeled separately to constrain models by physiology and by a number of total model variables.

### Secondary category of ML models

#### Task-related EEG source network power + task performance

These models tested whether adding task performance would improve the cortical source models. There were 12 relevant datasets in this category duplicated from the primary category and excluded the rest. Each of the IC, IP, and EB task models added d’ signal detection sensitivity, RT, and consistency variables for a total of 35 variables per model and the WM task additionally had the item span variable for a total of 36 variables per model.

#### Event-related EEG source network power

These models tested whether cognitive events (cue, stim, resp, fdbk) regardless of task can better predict SI than task-specific modeling as in the primary category. Hence, these datasets were split based on cognitive events. There were a total of 12 datasets in this category corresponding to 4 cognitive events for each of the 3 frequency bands. Each dataset included power from the 4 cognitive tasks (IC, IP, WM, EB) in all 8 brain networks (32 variables/model).

#### Task-related EEG scalp power

These models tested the performance of scalp EEG-based predictors. There were 12 datasets in this category in the same format as the primary dataset excluding the rest, except we had 7 scalp electrode groups instead of the 8 source brain networks (Table S2). This also serves as a good reference point to highlight the utility of the primary category since there is no new measurable information being added by source localization.

A full breakdown of all primary and secondary datasets and variables used can be found in Table S3.

Each dataset was tested independently with the goal of identifying a domain with the greatest predictive power for SI+/− classification. The reason we did not use more traditional forms of dimensionality reduction like principal component analysis (PCA) is so that we may be able to biologically interpret the predictive models. Likewise, brute forcing all combinations of variables is also not desirable due to the computational limitations of running that many models, in addition to the interpretability of the best model.

Notably, since we have a limited dataset, all datasets were finalized before any model was built based on our own a priori hypothesis on which conditions may present the greatest contrast between the two groups as per the categories described above. As such, all these datasets were created before any models were finalized so there is no risk of data leakage from this stage. We avoided using any ML methods for the purposes of feature reduction or dataset curation as that can also lead to model overfitting [61].

### ML pipeline

A robust ML pipeline was created using Python 3 and scikit-learn library to test each dataset independently to find the best-fit model that can accurately classify SI+ from SI-. The pipeline contained 3 major steps to preserve data integrity throughout all the tests:

- **Preprocessing ML models:** To handle any missing data from subjects missing certain neural task data, we used iterative imputation [62]. Only 2.3% of data points were imputed in the event-related EEG source network power datasets that collated data across all cognitive tasks. Other datasets were task-specific so subjects missing the task would be completely removed from the dataset. Following this, a standard scaler was applied to the dataset before entering the model training. Importantly, these stages (including the actual model training) were wrapped as a “Python pipeline object”. There are multiple benefits to this method, but the key benefit is avoiding leaking information on the distribution of the training set onto the testing set during cross-validation (CV).
- **Choosing Models and Parameters:** For this study, we decided to go with 3 different architectures, increasing in complexity. The first is the Logistic Regression (LR) model as a baseline simpler model, then a Decision Tree (DT) since it can be easily interpreted, and finally a multi-layer perceptron (MLP) model for a deeper neural network to be able to capture any non-linear dynamics from the datasets.
- **Nested CV and Hyper-Parameter Tuning:** Due to having a limited dataset, and in order to limit model overfitting and bias during the hyper-parameter tuning, we opted to use a repeated five-fold nested CV approach for assessing model accuracy. Although this nested approach is not the same as having an independent testing set, each of the outer 5 folds is treated as having a train/test/validation split. The first split of 80/20 leaves the 20 as the validation split, and the 80 is further split into train/test with the inner 5 folds. The result is that each of the 5 outer loops is validated on data that was never used in the training of the model at any point. More details on the nested CV algorithm are provided in **Supplementary Materials and Methods** [52, 63].

A simplified flowchart of the entire process can be seen in Fig. 2A.

### Metrics

The ML training was optimized for Matthew’s correlation coefficient (MCC) which measures the difference between the predicted values and actual values and is equivalent to the $\chi$^2^ statistics for a 2 × 2 contingency table [64]. We opted for MCC as it can be advantageous over other metrics such as the F1 score for model performance because it is both invariant to class swapping and has a high value (close to 1) when all quadrants of the confusion matrix perform well. We also computed sensitivity (SEN) and specificity (SPE) at each phase to offer a more comprehensive understanding of model performance. We further report the mean and std of these metrics across the nested CV to give a full representation of both type 1 and 2 errors across our models. Given that our datasets were generally balanced with equivalent data across SI+/−, barring a few missing data points from one or two subjects, we were not concerned about overfitting to a dominant class. A naïve model will have MCC, SEN, and SPE scores of 0, 0.5, and 0.5, which served as our baseline.

### Feature importance

In addition to identifying a successful dataset that can classify SI, we further identified the feature importance of individual variables within the best-performing model. This gave us more insight into the exact processes driving the best-fit model. Feature importance scores were calculated using SHapley Additive exPlanations (SHAP) [52, 65, 66]. The top 5 features of the best model were plotted in raincloud plots and group-wise t-tests were also calculated for these variables. This allowed us to visualize the separation and verify that the two groups indeed have some data-wise separation, increasing confidence that the results we found are valid.

### Independent validation with the blind dataset

As a final step to assess the generalizability of the best model, we collated an external validation dataset of 35 subjects with clinically-diagnosed depression that also performed the *BrainE* neuro-cognitive assessments. In this sample, the neuro-cognitive assessments were implemented in a modified version, i.e., were adaptive such that the response window on each task trial was adjusted with a 3up-1down staircase scheme that maintains accuracy at ∼80% and engages the user by avoiding ceiling performance [67]. An adaptive scheme reduces practice effects that affect repeat assessment sessions and was implemented because these subjects were part of a repeat-assessment study. Their baseline neuro-cognitive session was used for external validation, and we applied the final best model obtained from the main community-based study participants above to these clinical subjects.

## Results

Behavioral performance for the four cognitive tasks is shown in Table 2, split by the two groups SI+/−. Signal detection sensitivity d’ (scaled to 1), response time, and consistency metrics are shown. Between-group comparisons across performance metrics and tasks were made using t-tests and false discovery rate (FDR)-corrections were applied for multiple comparisons. We found no behavioral differences between SI+/− group task performance.

**Table 2:** Behavioral performance across the four cognitive tasks.

Cognitive Task	SI + mean ± sem	SI- mean ± sem	Effect Size, *p*-value
Inhibitory Control (IC)			
scaled d’	0.83 ± 0.03	0.84 ± 0.03	−0.09, 0.85
Wait response time	2.24 ± 0.03	2.13 ± 0.05	0.79, 0.27
Wait consistency	0.81 ± 0.03	0.73 ± 0.04	0.68, 0.35
Interference Processing (IP)			
Scaled d	0.78 ± 0.03	0.72 ± 0.03	0.32, 0.44
Response time	0.48 ± 0.02	0.50 ± 0.02	-0.18, 0.80
Consistency	0.85 ± 0.01	0.82 ± 0.02	0.32, 0.44
Working Memory (WM)			
Scaled d’	0.44 ± 0.04	0.41 ± 0.03	0.12, 0.85
Response time	0.48 ± 0.03	0.44 ± 0.02	0.25, 0.61
Consistency	0.77 ± 0.01	0.72 ± 0.01	0.64, 0.09
Item span	0.63 ± 0.05	0.66 ± 0.06	−0.08, 0.85
Emotion Bias (EB)			
Scaled d’	0.72 ± 0.02	0.73 ± 0.02	−0.06, 0.85
Response time	0.50 ± 0.02	0.50 ± 0.02	−.02, 0.94
Consistency	0.80 ± 0.01	0.80 ± 0.01	0.11, 0.85

Mean ± standard error of the mean (sem) data are shown for individuals with suicidal ideation (SI+, *n* = 38) vs. no suicidal ideation (SI-, *n* = 38). d’ sensitivity is scaled to 1. Response time is reported in seconds and response consistency is 1 – coefficient of variation of response time. The working memory span was tested for up to 8 items and is scaled to 1. Cohen’s d effect sizes are shown with positive values denoting higher scores in SI+ and negative values denoting higher scores in SI-. p-values represent FDR-corrected t-tests for group comparisons across all 13 variables; there were no statistically significant performance differences that survived multiple comparisons.

**Fig. 3:**
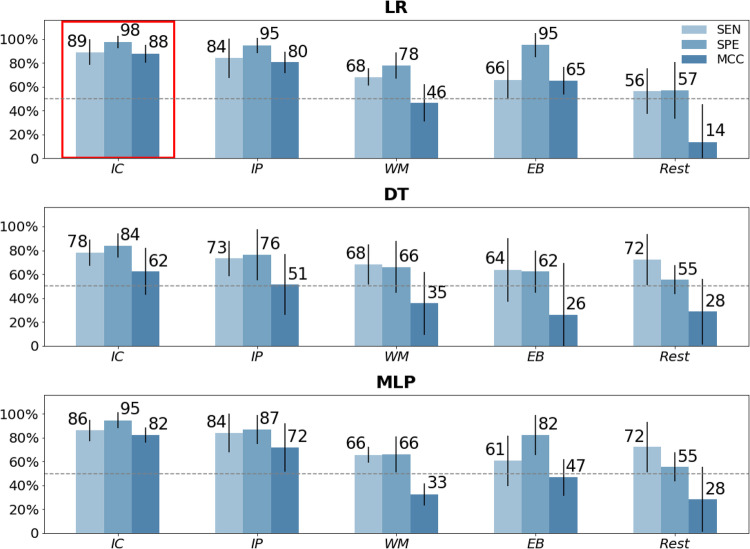
Performance of ML models for classifying suicidality across cognitive tasks. Average sensitivity (SEN), specificity (SPE), and Matthews Correlation Coefficient (MCC) scores are shown as percentages across nested 5-fold cross-validation run plotted with standard deviation. Results are shown for beta band models that were best performing. Performance metrics are shown in the three rows for the three types of models, LR Logistic Regression, DT Decision Tree, and MLP Multi-layer Perceptron, and shown in each row for the four tasks, IC Inhibitory Control, IP Interference Processing, WM Working Memory, EB Emotion Bias and Rest: resting state. The red box annotates the best-performing model by MCC score and average of sensitivity and specificity. The dashed grey line indicates a random chance model.

The overarching ML pipeline and the 8 brain networks modeled in cortical source space are shown in Fig. 2. The ML pipeline was executed for all primary and secondary category datasets defined in the Methods. Overall, beta band power in our primary category of models, i.e., cognitive task-related EEG source network power, specifically in the Inhibitory Control (IC) task proved to be best-performing by MCC score (of 88%) and average sensitivity and specificity (of 93.5%). The full results across all datasets in this category across the three ML models (LR, DT, and MLP) are shown in Fig. 3. Mean and standard deviation are plotted for each of the 3 metrics, calculated from the 5-fold outer CV loops. The best results are from beta power during the IC task using logistic regression boxed in red. We additionally tested if the IC model is improved by just including one type of stimulus condition, either the Go fast response trials or the Wait to respond trials, but separating these trial types did not improve the classification. Corresponding theta and alpha frequency band models in the primary category are shown in Figs. S1 and S2.

Further, we compared the best results from the primary models with the secondary model categories; comparisons are shown in Table 3. These results confirmed our primary hypothesis that cognitive task-related EEG source imaging may generate the best classifier. In this case, the LR model for the IC task-related beta source power was the best of all models based on the MCC score and average of sensitivity and specificity. Of note, the cognitive event-related source power MLP model, specifically for alpha band power during the response period (including data for all four cognitive tasks in the model) was the next best model, and models in source space performed much better than models in scalp space. The best dataset in each cognitive task/event-related model category performed better than both chance (SEN/SPE = 50%, MCC = 0) as well as the resting state dataset [64]. We are also confident that the results shown are not due to false positives/negatives since those would manifest as low specificity/sensitivity, respectively.

**Table 3:** Best performing ML model comparisons by category of models.

Model Category	Best Model: Frequency: Dataset	Sensitivity	Specificity	MCC
Task related EEG Source Network Power	LR: beta: IC	89 ± 11	98 ± 5	88 ± 8
Task related EEG Source Network Power + Task Performance	LR: beta: IC	89 ± 13	92 ± 10	81 ± 23
Event related EEG Source Network Power	MLP: alpha: Response	87 ± 16	100 ± 0	88 ± 15
Task related EEG Scalp Power	LR: beta: WM	78 ± 15	55 ± 17	35 ± 23

We compared the best-performing ML model for cognitive task-related EEG source network power observed in Fig. 3 (top row) with the best-performing models when including behavioral performance data (second row), or when organizing the datasets as cognitive event-related source network power (third row), or task-related scalp power (last row). Separate models were generated for power in theta (4–8 Hz), alpha (8–12 Hz), and beta (13–30 Hz) frequency bands. *LR* Logistic Regression, *MLP* Multi-layer Perceptron, *IC* Inhibitory Control.

**Fig. 4:**
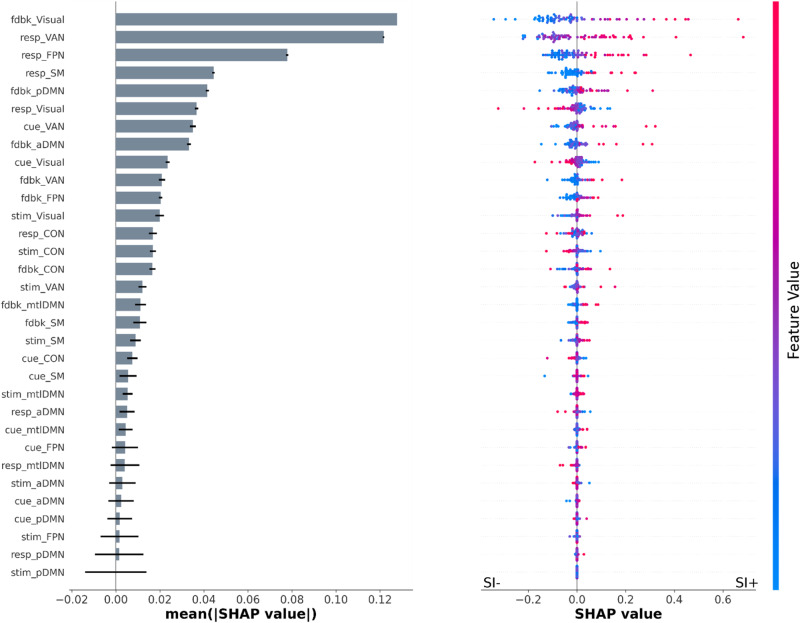
Rank and directionality of features predicting suicidal ideation in the best-performing classification model. On the left, all features predicting the SI+/− classification in the best performing LR: Beta: IC model, ranked by average Shapley value shown with standard errors for 5-folds of cross-validation. Feature names are coded by a cognitive event within the task followed by power in a specific neural network: cue, stim, resp, or fdbk. At the right, Shapley dot plots of all ranked feature predictors show the directionality of prediction; each dot represents a single datapoint, red dots indicate larger positive feature values while blue dots indicate larger negative feature values. X axis is the Shapley values with a center point at 0. Positive Shapley values increase the model output (closer to 1 or SI+), and negative Shapley values decrease the model output (closer to 0 or SI−).

We used Shapley statistics to determine the top predictors of SI classification in the best-fit model. The Shapley values for the best performing LR: beta: IC model are shown in Fig. 4. On the left we plot the feature importance of each variable with standard deviation shown for the 5-fold cross-validation runs. The variable names for feature importance are in the format of “cognitive event”_”brain network”. The variables are ranked by the mean absolute shapley values for each. The plot on the right shows the feature effects in the model, where each colored point represents a single data point for each feature. Dense areas on the plot are jittered to show the distribution of points. Each row corresponds to the respective variable shown on the left.

**Fig. 5:**
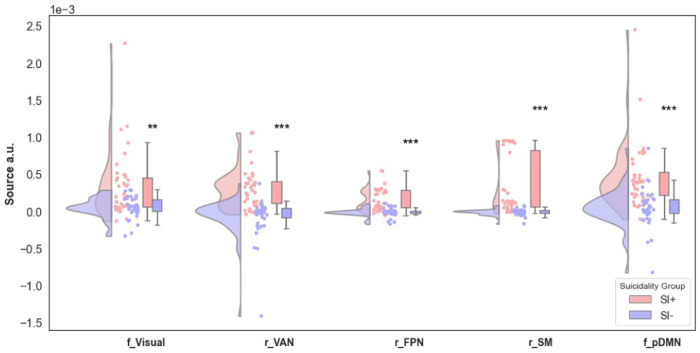
Raincloud plots for the top 5 predictors in the best-fit model, with the most important variable on the left going towards the right. Distribution, scatterplot, and box plots are shown. SI- is shaded blue and SI+ is shaded red. Y-axis is in EEG source power arbitrary units. FDR-corrected *p*-values, **p < 0.01, ***p < 0.001. All significances remain after removing outlier points defined as >3 z-scores within their group.

As we observed the feature importance of the predictors to decline after the first few variables per Fig. 4, we investigated the distribution of the top five predictors using raincloud plots (Fig. 5). These plots showed significant differences in the distribution of beta source power for all five top variables comparing SI+ vs. SI− groups. The top predictor variables were feedback period beta power in the Visual network, response period beta power in two cognitive control networks (Ventral Attention Network and Frontal Parietal Network) as well as the sensory-motor network, and feedback period beta power in the posterior DMN; in all cases, the SI+ group showed greater power than the SI- group.

Finally, we tested the external validity of the best model, LR:beta:IC with an independent clinical dataset. For 35 subjects (23 SI−, 12 SI+) we obtained sensitivity, specificity, and MCC metrics as 50%, 61%, and 0.1 respectively. It is possible that the modified, i.e. adaptive IC assessment applied in this clinical dataset led to suboptimal sensitivity/specificity, or that in general there is a limit to generalizability to independent clinical data. The best model in the secondary category (MLP:alpha:response) did not perform better than the best primary model at classifying the clinical dataset.

## Discussion

In this study, we aimed at classifying suicidal ideation based on neural predictors obtained during cognitive processes, within a balanced SI+/− cohort matched for potentially confounding variables such as depression and anxiety. We successfully demonstrated an accurate classifier for suicidal ideation (overall accuracy = 93%, percentage of correct vs. total predictions), obtained using EEG source imaging data during the inhibitory control task. We additionally isolated the specific neural predictors to gain insights into how SI changes brain activity.

Across spectral power in the three frequency bands (theta, alpha, beta), the best-performing ML model was obtained with cortical source-localized beta band power measured during the IC task. Several previous studies have linked beta band spectral activity changes into neuropsychiatric disorders, like anxiety, depression, and suicidality [68, 69]. Past literature has also affirmed that individuals engaging in self-harm commit increased errors in the IC task, suggesting that there may be altered neural processes going on as well [32].

When inspecting the top predictors in this best-fit model, we observed elevated beta band power within posterior visual/default-mode-network regions during the feedback period as well as elevated beta power within attention networks (both ventral and fronto-parietal) and sensorimotor regions during the response period linked with a higher probability of being classified as SI+ (as seen in Fig. 4). Beta oscillations have been linked with a number of higher-order cognitive functions including attention, working memory and executive function [70–72]. There is particularly a body of work linking prefrontal [73, 74] and sensorimotor beta oscillations [75] with inhibition. Notably, prior work on a stop signal task demonstrated higher levels of beta oscillations specifically in right inferior frontal cortex (part of the ventral attention network, as we observe here) are linked with improved inhibition [73, 74]. It is important to note that performance on this inhibitory control task was not different between individuals with and without SI – and thus, the increased power in these circuits likely reflects a compensatory increase in power required to maintain a similar level of behavioral performance on this task, as has been suggested previously [76]. Consistent with this, we also did not find cognitive performance differences between SI+/− individuals. Further, our best-fit model performance dropped when adding in the task performance variables, suggesting that observed differences between SI+/− individuals are primarily driven by alterations in neural physiology rather than explicit behavior. This corresponds to other work suggesting that suicidality might impair executive control in brain networks contributing to the physiological alterations observed in the inhibitory control task [77]. Thus, executive control dysfunction may be reflected not in impaired task behavioral performance but instead in altered frontal cortical activity that serves as a salient marker for suicidal behavior [78]. Feedback-related modulation of beta oscillations has also been previously implicated in adaptive task performance [79, 80]. Prior work has further shown that altered beta band activity within parieto-occipital regions is responsible for forming one’s sense of agency [81]. These impairments could contribute to the external locus of control and a distorted sense of self-agency often observed in individuals at risk for suicide [82].

Notably, our study was able to achieve an accurate prediction of SI using a larger sample size than previous EEG and fMRI studies [13, 83], with the exception of some neuroimaging studies that had larger sample sizes [12, 84]. Since we used matched groups that controlled for potentially confounding factors like demographics as well as mental health symptoms of depression and anxiety, we can be more confident the model is acting on the main target of suicidal ideation vs. no suicidal ideation as opposed to other symptoms that may be related to SI. The model was also developed using variables that are meaningful in the field of neuroscience and psychiatry instead of a purely mathematical model optimized for the highest accuracy. The combination of these two factors allows us to suggest potential cognitive neural markers for suicidal ideation. In addition, given the success of the modeling in this study, the streamlined and rapid cognitive task structure, and the scalability of EEG as a modality to measure the power of brain oscillations, there is high utility in adopting these methods for replication in the future.

Our study has some identifiable limitations. First, although our sample size was larger than some previous studies [13, 83], incorporating more subjects (*N* > 500) and a separate clinical dataset to test on that has implemented the exact same assessments would help increase confidence in the specific regions of the brain that impact suicidal ideation and provide more clinical specificity for targeted therapeutics. Additionally, the data used for this model only came from a single setting which may not generalize to other settings. In a recent study [85], ML models aimed at predicting treatment success for schizophrenia found that models built on one dataset (including both single-trial and multi-trial data sets) did not generalize to other independent studies. In that study, the lack of generalization may be limited by several factors, however. First: only 3 out of the 5 single trial models built by the authors performed above chance and had a balanced accuracy of around 60%. Low accuracy within trial models will inherently be poor at generalization. Second: schizophrenia reflects a heterogenous cluster of symptoms that is better defined as a syndrome rather than a biological entity [86]. Only one of the studies mentioned previously implemented an external validation set using fMRI, so the generalizability of EEG-built models is still currently unknown [83]. Our replication with an external clinical dataset shows the difficulties of building a generalizable model as well. However, there were also methodological differences in our external validation sample in that they completed performance-adaptive assessments with an inherently reduced number of trials, which may lead to noisier data. In addition, our original sample size is not large enough such that it would produce a robust and highly generalizable model. Despite these shortcomings, we still believe the pipeline has the potential to allow us to better understand the physiological implications of suicidal ideation tied to cognitive performance. With larger sample sizes we believe that a generalizable model is achievable.

Other limitations of our study are utilizing limited EEG features, i.e., spectral power only, and utilizing simpler ML models. Incorporating additional EEG temporal dynamic features and more complex ML techniques may allow for more informative models/ reveal additional neural biomarker predictors, but we did not explore these options given the high accuracy we obtained with our base models. Future studies could also selectively decrease or increase the number of EEG channels recorded to either streamline the data collection process or increase the spatial sensitivity of brain regions. Although there were no significant ethnic differences between the two groups, the prevalence of suicidal ideation does differ across minoritized groups which could be a confound [87, 88]. In the case of gender, both groups were majority female so we are less concerned with this imbalance. Lastly, the ML models in this study were built to differentiate individuals with and without suicidal ideation in a community sample, outside a formal clinical setting. While this acts as a useful tool to mitigate the imminent risk of suicide, future studies should test this model to predict other aspects of suicidal behavior as well as continue to validate how well this model translates to a clinical setting.

Overall, our research showcases the capability of cognitive task-linked EEG source imaging for predicting suicidality and identifies relevant neural biomarkers involved in risk prediction. Additionally, the study opens several new investigation avenues in terms of leveraging scalable brain mapping methods to serve this vulnerable clinical population.

### Supplementary information

Supplemental Material

## References

[1] Prevention C for DC and. Centers for Disease Control and Prevention. 2023;2023. https://www.cdc.gov/suicide/suicide-data-statistics.html.

[2] O'Rourke MC, Jamil RT, Siddiqui W. Suicide Screening and Prevention. 2023 Mar 6. In: StatPearls [Internet]. Treasure Island (FL): StatPearls Publishing; 2024. 30285348

[3] Vilhjalmsson R, Kristjansdottir G, Sveinbjarnardottir E (1998). Factors associated with suicide ideation in adults. Soc Psychiatry Psychiatr Epidemiol.

[4] Yan Y, Hou J, Li Q, Yu NX (2023). Suicide before and during the COVID-19 Pandemic: A systematic review with meta-analysis. Int J Environ Res Public Health.

[5] Aldhyani THH, Alsubari SN, Alshebami AS, Alkahtani H, Ahmed ZAT (2022). Detecting and analyzing suicidal ideation on social media using deep learning and machine learning models. Int J Environ Res Public Health.

[6] Liu J, Shi M, Jiang H (2022). Detecting suicidal ideation in social media: An ensemble method based on feature fusion. Int J Environ Res Public Health.

[7] Roy A, Nikolitch K, McGinn R, Jinah S, Klement W, Kaminsky ZA (2020). A machine learning approach predicts future risk to suicidal ideation from social media data. NPJ Digit Med.

[8] Navarro MC, Ouellet-Morin I, Geoffroy MC, Boivin M, Tremblay RE, Cote SM (2021). Machine learning assessment of early life factors predicting suicide attempt in adolescence or young adulthood. JAMA Netw Open.

[9] Su R, John JR, Lin PI (2023). Machine learning-based prediction for self-harm and suicide attempts in adolescents. Psychiatry Res.

[10] Sudol K, Mann JJ (2017). Biomarkers of suicide attempt behavior: towards a biological model of risk. Curr Psychiatry Rep.

[11] Chang BP, Franklin JC, Ribeiro JD, Fox KR, Bentley KH, Kleiman EM (2016). Biological risk factors for suicidal behaviors: a meta-analysis. Transl Psychiatry.

[12] Bajaj S, Blair KS, Dobbertin M, Patil KR, Tyler PM, Ringle JL (2023). Machine learning based identification of structural brain alterations underlying suicide risk in adolescents. Discov Ment Health.

[13] Hasey G, Reilly J, Colic S, Maccrimmon D, Rostamabad AK, Debruin H. Detection of suicidal ideation in depressed subjects using resting electroencephalography features identified by machine learning algorithms. Biol Psychiatry. 2020;87:S380–1.

[14] Schmaal L, van Harmelen A-L, Chatzi V, Lippard ETC, Toenders YJ, Averill LA (2020). Imaging suicidal thoughts and behaviors: a comprehensive review of 2 decades of neuroimaging studies. Mol Psychiatry.

[15] Bohaterewicz B, Sobczak AM, Podolak I, Wojcik B, Metel D, Chrobak AA (2020). Machine learning-based identification of suicidal risk in patients with schizophrenia using multi-level resting-state fMRI features. Front Neurosci.

[16] Wang Q, He C, Wang Z, Fan D, Zhang Z, Xie C (2023). Connectomics-based resting-state functional network alterations predict suicidality in major depressive disorder. Transl Psychiatry.

[17] Keilp JG, Grunebaum MF, Gorlyn M, LeBlanc S, Burke AK, Galfalvy H (2012). Suicidal ideation and the subjective aspects of depression. J Affect Disord.

[18] Purselle DC, Heninger M, Hanzlick R, Garlow SJ (2009). Differential association of socioeconomic status in ethnic and age-defined suicides. Psychiatry Res.

[19] Wu W, Zhang Y, Jiang J, Lucas MV, Fonzo GA, Rolle CE (2020). An electroencephalographic signature predicts antidepressant response in major depression. Nat Biotechnol.

[20] Lee Y, Ragguett R-M, Mansur RB, Boutilier JJ, Rosenblat JD, Trevizol A (2018). Applications of machine learning algorithms to predict therapeutic outcomes in depression: A meta-analysis and systematic review. J Affect Disord.

[21] Patel MJ, Andreescu C, Price JC, Edelman KL, Reynolds CF, Aizenstein HJ (2015). Machine learning approaches for integrating clinical and imaging features in late‐life depression classification and response prediction. Int J Geriatr Psychiatry.

[22] McGrath CL, Kelley ME, Holtzheimer PE, Dunlop BW, Craighead WE, Franco AR (2013). Toward a neuroimaging treatment selection biomarker for major depressive disorder. JAMA Psychiatry.

[23] Dunlop BW, Mayberg HS (2014). Neuroimaging-based biomarkers for treatment selection in major depressive disorder. Dialogues Clin Neurosci.

[24] Athreya AP, Neavin D, Carrillo‐Roa T, Skime M, Biernacka J, Frye MA (2019). Pharmacogenomics‐driven prediction of antidepressant treatment outcomes: a machine‐learning approach with multi‐trial replication. Clin Pharm Ther.

[25] Bankwitz A, Rüesch A, Adank A, Hörmann C, Villar de Araujo T, Schoretsanitis G (2023). EEG source functional connectivity in patients after a recent suicide attempt. Clin Neurophysiol.

[26] Lee SM, Jang K-I, Chae J-H (2017). Electroencephalographic correlates of suicidal ideation in the Theta band. Clin EEG Neurosci.

[27] Arikan MK, Gunver MG, Tarhan N, Metin B (2019). High-Gamma: A biological marker for suicide attempt in patients with depression. J Affect Disord.

[28] Butler LB, Nooner KB (2023). The link between suicidality and Electroencephalography asymmetry: a systematic review and meta-analysis. Clin Psychopharmacol Neurosci.

[29] He X-Q, Hu J-H, Peng X-Y, Zhao L, Zhou D-D, Ma L-L (2024). EEG microstate analysis reveals large-scale brain network alterations in depressed adolescents with suicidal ideation. J Affect Disord.

[30] Pu S, Setoyama S, Noda T (2017). Association between cognitive deficits and suicidal ideation in patients with major depressive disorder. Sci Rep.

[31] Allen KJ, Hooley JM (2015). Inhibitory control in people who self-injure: evidence for impairment and enhancement. Psychiatry Res.

[32] Porteous M, Tavakoli P, Campbell K, Dale A, Boafo A, Robillard R (2023). Emotional modulation of response inhibition in adolescents during acute suicidal crisis: event-related potentials in an emotional Go/NoGo task. Clin EEG Neurosci.

[33] Richard-Devantoy S, Berlim MT, Jollant F (2014). A meta-analysis of neuropsychological markers of vulnerability to suicidal behavior in mood disorders. Psychol Med.

[34] Grennan GK, Ramanathan DS, Mishra J, Withers MC. Differences in interference processing and frontal brain function with climate trauma from California’s deadliest wildfire. PLOS Climate. 2023;2:e0000125.

[35] Mo Z, Grennan G, Kulkarni A, Ramanathan D, Balasubramani PP, Mishra J (2023). Parietal alpha underlies slower cognitive responses during interference processing in adolescents. Behav Brain Res.

[36] Bridgett DJ, Oddi KB, Laake LM, Murdock KW, Bachmann MN (2013). Integrating and differentiating aspects of self-regulation: effortful control, executive functioning, and links to negative affectivity. Emotion.

[37] Kasper LJ, Alderson RM, Hudec KL (2012). Moderators of working memory deficits in children with attention-deficit/hyperactivity disorder (ADHD): a meta-analytic review. Clin Psychol Rev.

[38] Lin L, Wang C, Mo J, Liu Y, Liu T, Jiang Y (2020). Differences in behavioral inhibitory control in response to angry and happy emotions among college students with and without suicidal ideation: An ERP study. Front Psychol.

[39] Richard-Devantoy S, Olie E, Guillaume S, Bechara A, Courtet P, Jollant F (2013). Distinct alterations in value-based decision-making and cognitive control in suicide attempters: toward a dual neurocognitive model. J Affect Disord.

[40] Kroenke K, Spitzer RL, Williams JB (2001). The PHQ-9: validity of a brief depression severity measure. J Gen Intern Med.

[41] Spitzer RL, Kroenke K, Williams JBW, Löwe B (2006). A brief measure for assessing generalized anxiety disorder: the GAD-7. Arch Intern Med.

[42] Boudreau B, Poulin C (2009). An examination of the validity of the Family Affluence Scale II (FAS II) in a general adolescent population of Canada. Soc Indic Res.

[43] Oquendo MA, Halberstam B, Mann JJ, First MB Standardized evaluation in clinical practice. American Psychiatric Press, Washington DC. 2003:103–29.

[44] Misra A, Ojeda A, Mishra J. BrainE: a digital platform for evaluating, engaging and enhancing brain function. Regents of the University of California Copyright SD2018-816. 2018.

[45] Balasubramani PP, Ojeda A, Grennan G, Maric V, Le H, Alim F (2021). Mapping cognitive brain functions at scale. Neuroimage.

[46] Balasubramani PP, Walke A, Grennan G, Perley A, Purpura S, Ramanathan D (2022). Simultaneous gut-brain electrophysiology shows cognition and satiety specific coupling. Sensors.

[47] Kato R, Balasubramani PP, Ramanathan D, Mishra J (2022). Utility of cognitive neural features for predicting mental health behaviors. Sensors.

[48] Nan J, Balasubramani PP, Ramanathan D, Mishra J (2022). Neural dynamics during emotional video engagement relate to anxiety. Front Hum Neurosci.

[49] Balasubramani PP, Diaz-Delgado J, Grennan G, Alim F, Zafar-Khan M, Maric V (2023). Distinct neural activations correlate with maximization of reward magnitude versus frequency. Cereb Cortex.

[50] Grennan G, Balasubramani PP, Alim F, Zafar-Khan M, Lee EE, Jeste DV (2021). Cognitive and neural correlates of loneliness and wisdom during emotional bias. Cereb Cortex.

[51] Grennan G, Balasubramani PP, Vahidi N, Ramanathan D, Jeste DV, Mishra J (2022). Dissociable neural mechanisms of cognition and well-being in youth versus healthy aging. Psychol Aging.

[52] Shah RV, Grennan G, Zafar-Khan M, Alim F, Dey S, Ramanathan D (2021). Personalized machine learning of depressed mood using wearables. Transl Psychiatry.

[53] Fakhraei L, Francoeur M, Balasubramani PP, Tang T, Hulyalkar S, Buscher N (2021). Electrophysiological correlates of rodent default-mode network suppression revealed by large-scale local field potential recordings. Cereb Cortex Commun.

[54] Fakhraei L, Francoeur M, Balasubramani P, Tang T, Hulyalkar S, Buscher N (2021). Mapping large-scale networks associated with action, behavioral inhibition and impulsivity. ENeuro.

[55] Kothe C, Medine D, Boulay C, Grivich M, Stenner T Lab Streaming Layer. https://labstreaminglayer.readthedocs.io/. 2019. 10.1162/IMAG.a.136 PMC12434378 40959706

[56] Ojeda A, Kreutz-Delgado K, Mishra J (2021). Bridging M/EEG source imaging and independent component analysis frameworks using biologically inspired sparsity priors. Neural Comput.

[57] Ojeda A, Wagner M, Maric V, Ramanathan D, Mishra J (2023). EEG source derived salience network coupling supports real-world attention switching. Neuropsychologia.

[58] Rodríguez-González V, Gómez C, Shigihara Y, Hoshi H, Revilla-Vallejo M, Hornero R (2020). Consistency of local activation parameters at sensor- and source-level in neural signals. J Neural Eng.

[59] Michel CM, He B (2019). EEG source localization. Handb Clin Neurol.

[60] Ojeda A, Kreutz-Delgado K, Mullen T (2018). Fast and robust Block-Sparse Bayesian learning for EEG source imaging. Neuroimage.

[61] Verstynen T, Kording KP (2023). Overfitting to ‘predict’ suicidal ideation. Nat Hum Behav.

[62] Buuren Svan, Groothuis-Oudshoorn K (2011). mice: Multivariate Imputation by chained equations in *R*. J Stat Softw.

[63] Cawley GC, Talbot NLC (2010). On over-fitting in model selection and subsequent selection bias in performance evaluation. J Mach Learn Res.

[64] Chicco D, Jurman G (2020). The advantages of the Matthews correlation coefficient (MCC) over F1 score and accuracy in binary classification evaluation. BMC Genomics.

[65] Rashed-Al-Mahfuz MD, Moni MA, Uddin S, Alyami SA, Summers MA, Eapen V (2021). A deep convolutional neural network method to detect seizures and characteristic frequencies using Epileptic Electroencephalogram (EEG) data. IEEE J Transl Eng Health Med.

[66] Lundberg SM, Lee S-I. A unified approach to interpreting model predictions. In: Guyon I, Luxburg U Von, Bengio S, Wallach H, Fergus R, Vishwanathan S, et al., editors. Adv Neural Inf Process Syst, vol. 30, Curran Associates, Inc.; 2017.

[67] Jaiswal S, Nan J, Purpura SR, Manchanda JK, Garcia-pak I, Ramanathan DS, et al. Mindfulness coaching with digital lifestyle monitoring enhances selective attention in medical scientists. MedRxiv. 2024:2024.01.04.24300716. 10.1371/journal.pone.0330290 PMC12431218 40938915

[68] Anijarv TE, Can AT, Gallay CC, Forsyth GA, Dutton M, Mitchell JS (2023). Spectral changes of EEG following a 6-week low-dose oral ketamine treatment in adults with major depressive disorder and chronic suicidality. Int J Neuropsychopharmacol.

[69] Bentley KH, Franklin JC, Ribeiro JD, Kleiman EM, Fox KR, Nock MK (2016). Anxiety and its disorders as risk factors for suicidal thoughts and behaviors: A meta-analytic review. Clin Psychol Rev.

[70] Briley PM, Liddle EB, Simmonite M, Jansen M, White TP, Balain V (2021). Regional brain correlates of beta bursts in health and psychosis: a concurrent electroencephalography and functional magnetic resonance imaging study. Biol Psychiatry Cogn Neurosci Neuroimaging.

[71] Cao L, Hu YM (2016). Beta rebound in visuomotor adaptation: still the status quo?. J Neurosci.

[72] Spitzer B, Haegens S. Beyond the status quo: a role for beta oscillations in endogenous content (Re)Activation. ENeuro. 2017;4:ENEURO.0170-17.2017. 10.1523/ENEURO.0170-17.2017 PMC5539431 28785729

[73] Muralidharan V, Aron AR, Schmidt R (2022). Transient beta modulates decision thresholds during human action-stopping. Neuroimage.

[74] Sundby KK, Jana S, Aron AR (2021). Double-blind disruption of right inferior frontal cortex with TMS reduces right frontal beta power for action stopping. J Neurophysiol.

[75] Rossiter HE, Davis EM, Clark EV, Boudrias M-H, Ward NS (2014). Beta oscillations reflect changes in motor cortex inhibition in healthy ageing. Neuroimage.

[76] Marzuk PM, Hartwell N, Leon AC, Portera L (2005). Executive functioning in depressed patients with suicidal ideation. Acta Psychiatr Scand.

[77] Ram D, Chandran S, Sadar A, Gowdappa B (2019). Correlation of cognitive resilience, cognitive flexibility and impulsivity in attempted suicide. Indian J Psychol Med.

[78] Thompson C, Ong ELC (2018). The association between suicidal behavior, attentional control, and frontal asymmetry. Front Psychiatry.

[79] HajiHosseini A, Hutcherson CA, Holroyd CB (2020). Beta oscillations following performance feedback predict subsequent recall of task-relevant information. Sci Rep.

[80] Yaple Z, Martinez-Saito M, Novikov N, Altukhov D, Shestakova A, Klucharev V (2018). Power of feedback-induced beta oscillations reflect omission of rewards: evidence From an EEG gambling study. Front Neurosci.

[81] Bu-Omer HM, Gofuku A, Sato K, Miyakoshi M (2021). Parieto-Occipital Alpha and low-Beta EEG power reflect sense of agency. Brain Sci.

[82] Moore JW, Ruge D, Wenke D, Rothwell J, Haggard P (2010). Disrupting the experience of control in the human brain: pre-supplementary motor area contributes to the sense of agency. Proc Biol Sci.

[83] Gao M, Wong NML, Lin C, Huang C-M, Liu H-L, Toh C-H (2023). Multimodal brain connectome-based prediction of suicide risk in people with late-life depression. Nat Ment Health.

[84] Weng J-C, Lin T-Y, Tsai Y-H, Cheok M, Chang Y-P, Chen V (2020). An autoencoder and machine learning model to predict suicidal ideation with brain structural imaging. J Clin Med.

[85] Chekroud AM, Hawrilenko M, Loho H, Bondar J, Gueorguieva R, Hasan A (2024). Illusory generalizability of clinical prediction models. Science.

[86] Kirkpatrick B, Buchanan RW, Ross DE, Carpenter WT (2001). A separate disease within the Syndrome of Schizophrenia. Arch Gen Psychiatry.

[87] Morrison LL, Downey DL (2000). Racial differences in self-disclosure of suicidal ideation and reasons for living: Implications for training. Cult Divers Ethn Minor Psychol.

[88] O’Keefe VM, Wingate LR, Cole AB, Hollingsworth DW, Tucker RP (2015). Seemingly harmless racial communications are not so harmless: racial microaggressions lead to suicidal ideation by way of depression symptoms. Suicide Life Threat Behav.

