# Supplementary material for: Neural activity during inhibitory control predicts suicidal ideation with machine learning
Source: NPP Digit Psychiatry Neurosci. 2024 Jul 8;2:10. doi: 10.1038/s44277-024-00012-x (PMC11230903; doi:10.1038/s44277-024-00012-x)
Supplement: Supplementary file 1 — Supplemental Material [file 44277_2024_12_MOESM1_ESM.docx]

**Supplementary Materials and Methods**

**Neuro-cognitive Assessment Tasks**

All four neuro-cognitive tasks had a standard trial structure of 500 msec central fixation “+” cue followed by the task-specific stimulus presented for task-specific duration and with a task-specific response window (**Figure 1**). All stimuli were presented in a shuffled order across trials. Response on every task trial was followed by standard response feedback for accuracy as a smiley or sad face emoticon, presented 200 msec post-response for 200 msec duration, followed by a 500 msec inter-trial interval (ITI).

**Inhibitory Control (IC)**. Participants accessed a game-like task named Go Wait modeled after the standard test of variables of attention [1,2]. In this simple two-block task, colored rockets were presented either in the upper/lower central visual field, presented for 100 msec post-fixation. Participants were instructed to respond to blue rocket targets as quickly as possible within a 700 msec response window (“go” target trials) and briefly withhold their response to distracting rockets of five other iso-luminant colors (brown, mauve, pink, purple, teal); responses were made on the distracting rocket trials after 2 sec when the fixation “+” cue flashed briefly again on the screen for 100 msec (“wait” non-target trials). Response was followed by emoticon feedback. Both task blocks lasted 5 minutes and consisted of 90 trials per block with 30/60 target/nontarget ratio in block 1 and 60/30 ratio in block 2.

**Interference Processing (IP)**. Participants accessed a game-like task named Middle Fish, an adaptation of the Flanker task [3], which has been extensively used to study interfering distractor processing [4,5]. Participants were instructed to respond to the direction of a centrally located target (middle fish) while ignoring all flanking distractor fish. On congruent trials the flanker fish faced the same direction as the central fish, while on incongruent trials they faced the opposite direction. 96 trials presented over two blocks for a total task time of 8 min. 50% of trials had congruent distractors and 50% were incongruent. To retain attention, the array of fish was randomly presented in the upper or lower visual field on equivalent number of trials. Stimulus duration was 100 msec with up to a 1 sec response window in which participants responded left/right as per the direction of the middle fish, followed by happy/sad emoticon feedback.

**Working Memory (WM)**. Participants accessed a game-like task named Lost Star that is based on the standard visuo-spatial Sternberg task [6]. Participants were presented a set of test objects (stars); they were instructed to maintain the visuo-spatial locations of the test objects in WM for a 3 sec delay period, and then responded whether a probe object (star) was or was not located in the same place as one of objects in the original test set. We implemented this task at the threshold perceptual span for each individual, i.e. the number of star stimuli that the individual could correctly encode without any WM delay. For this, a brief perceptual thresholding period preceded the main working memory task, allowing for equivalent perceptual load to be investigated across participants[4]. During thresholding, the set size of the test stars was progressively increased from 1-8 stars based on accurate performance; 4 trials were presented at each set size and 100% performance accuracy led to an increment in set size; <100% performance led to one 4-trial repeat of the same set size and any further inaccurate performance aborted the thresholding phase. The final set size at which 100% accuracy was obtained was designated as the individual's perceptual threshold. Post-thresholding, the WM task consisted of 48 trials presented over 2 blocks[7]. Post-fixation cue on each trial, test star objects were presented for 1 sec followed by a 3 sec WM delay period followed by a single probe star object for 1 sec, and finally a response time window of up to 1 sec in which participants made a yes/no choice whether the probe star had a matching location to the previously presented test set. Response was followed by happy/sad emoticon feedback. The total task duration was 6 min.

**Emotion Bias (EB)**. Participants accessed a game-like task named Face Off adapted from studies of attentional bias in emotional contexts [8–10]. The task integrated a standardized set of culturally diverse faces from the NimStim database [11]. We used an equivalent number of male and female faces, each face with four sets of emotions, either neutral, positive (happy), negative (sad) or threatening (angry), presented on equivalent number of trials. Post-fixation cue on each trial, participants viewed an emotional face with a superimposed arrow of 300 msec duration. The arrow occurred in either the upper or lower central visual field on equal number of trials, and participants responded to the direction of the arrow (left/right) within an ensuing 1 sec response window. Participants completed 144 trials presented over three equipartitioned blocks with shuffled emotion trials in each block. The total task duration was 10 min.

**Rest.** Participants were instructed to simply close their eyes and rest for ~3 minutes which served as a resting state baseline.

**Behavior Analysis**

Behavioral data for all cognitive tasks were analyzed for signal detection sensitivity, d’, computed as z(Hits)-z(False Alarms) [12]. All d’ values were divided by max theoretical d’ of 4.65 to obtain scaled d’ in the 0–1 range. Response times (RT) from stimulus onset are measured in seconds. Task consistency is also measured, defined as 1- (standard deviation of response times/mean response times). For the working memory task, perceptual span was also calculated.

**EEG Preprocessing**

Step 1) EEG channel data processing was conducted using the EEGLAB toolbox v2020 in MATLAB v2021a[13]. EEG data was resampled at 250 Hz and filtered in the 1-45 Hz range to exclude ultraslow DC drifts at <1Hz and high-frequency noise produced by muscle movements and external electrical sources at >45Hz.

There were no missing channels in the EEG data across subjects. Epoched data were cleaned using the autorej function in EEGLAB to remove noisy trials, i.e. >5SD outliers rejected over max 8 iterations, followed by further cleaning of electrooculographic, electromyographic or non-brain source artifacts using the Sparse Bayesian learning (SBL) algorithm (https://github.com/aojeda/PEB) [14,15].The cleaned data were then band filtered in the physiologically relevant theta (4-8 Hz), alpha (8-13 Hz), and beta (13-30 Hz) frequency bands. Our experimental setup was not in a very low-noise/shielded environment to allow for gamma band analyses, hence, this frequency band was not included in the analyses. Epoched events locked to cue, stimulus, response and feedback in each cognitive task were then extracted and averaged across trials to remove single trial noise. Epoched data were baseline corrected relative to the -750 msec to -550 msec time window prior to stimulus presentation in each task and electrode in each subject[16]. Since resting state is not in a trial-like structure, we artificially create trials by dividing the signal into equal windows, each 4 seconds long and time-locked to pseudorandom time points during rest.

Step 2) We used the block-Sparse Bayesian learning (BSBL-2S) algorithm to localize frequency band filtered EEG data and partitioned the signals into cortical regions of interest (ROIs) and artifact sources [14,15]. For the source space activations, ROIs were based on the standard 68 brain region Desikan-Killiany atlas [17] using the Colin-27 head model [18]. BSBL-2S is a two-step algorithm in which the first-step is equivalent to low-resolution electromagnetic tomography (LORETA [19]). LORETA estimates sources subject to smoothness constraints, i.e. nearby sources tend to be co-activated, which may produce source estimates with a high number of false positives that are not biologically plausible. To guard against this, BSBL-2S applies sparsity constraints in the second step wherein blocks of irrelevant sources are pruned. Notably, this data-driven sparsity constraint reduces the effective number of sources considered at any given time as a solution. The sparsity is imposed at the level of cortical ROIs, thereby projecting the data onto this space of few ROIs, and reducing the uncertainty of the inverse solution. Thus, it is not that only higher channel density data can yield source solutions, the ill-posed inverse problem can also be solved by imposing more aggressive constraints on the solution to converge on the source model at lower channel densities, as also supported by prior research [20,21]. Of note, the BSBL-2S two-stage algorithm has been benchmarked to produce evidence-optimized inverse source models at 0.95AUC relative to the ground truth, while without the second stage <0.9AUC is obtained, verified using both data and simulations [14,15]. We have also shown that cortical source mapping with this method has high test-retest reliability (Cronbach’s alpha = 0.77, p<0.0001) obtained with recordings conducted one-week apart[16]. Population outliers across all subjects’ source data were removed using the >5SD criterion.

**Machine Learning: Nested CV:** This algorithm is used to perform hyperparameter tuning and model selection by attempting to overcome the problem of overfitting the training dataset. It involves treating model hyperparameter tuning as part of the model itself and evaluating it within the broader k-fold CV procedure for evaluating models for comparison and selection. The k-fold CV procedure for model hyperparameter optimization is nested inside the k-fold CV procedure for model selection. Given that the procedure uses two CV loops it is also called double CV. Typically, the k-fold CV procedure involves fitting a model on all folds but one and evaluating the fit model on the holdout fold. Nested CV estimates the generalization error of the underlying model and its hyperparameter search. By choosing the parameters that maximize non-nested CV, biases the model to the dataset, yielding an overly optimistic score. Model selection without nested CV uses the same data to tune model parameters and evaluate model performance. Information may thus "leak" into the model and overfit the data. The magnitude of this effect is primarily dependent on the size of the dataset and the model's stability. To avoid this problem, nested CV effectively uses a series of train/validation/test set splits. In the inner loop, the score is approximately maximized by fitting a model to each training set and then directly maximized in selecting hyperparameters over the validation set. In the outer loop, generalization error is estimated by averaging test set scores over several dataset splits. Under this procedure, the hyperparameter search does not have an opportunity to overfit the dataset as it is only exposed to a subset of the dataset provided by the outer CV procedure. This reduces, if not eliminates, the risk of the search procedure overfitting the original dataset and should provide a less biased estimate of a tuned model’s performance on the dataset. In this way, the performance estimate includes a component properly accounting for the error introduced by overfitting the model selection criterion. As far as model selection is concerned, a nested CV scheme produces k-surrogate “best” models out of which one has to be chosen based on a stability measure, which will then be refitted onto the dataset for intervention purposes. A repeated k-fold CV repeats k-fold n times with different randomization in each repetition. This ensures the robustness and stability of the model. We used a repeated 5-fold CV scheme with five repeats as the inner CV strategy and a simple 5-fold CV scheme as the outer CV strategy for the overall nested CV scheme. The overall best model selection of the five surrogate best models that the inner CV scheme produces is made and refitted over a repeated 5-fold CV[22].

**Supplementary Tables**

| **Network** | **ROI** | **Network** | **ROI** |
| --- | --- | --- | --- |
| **Fronto-Parietal Network (FPN)** | caudalmiddlefrontal L | **medial temporal lobe Default Mode Network (mtlDMN)** | entorhinal L |
|  | caudalmiddlefrontal R |  | entorhinal R |
|  | rostralmiddlefrontal L |  | inferiortemporal L |
|  | rostralmiddlefrontal R |  | inferiortemporal R |
|  | superiorparietal L |  | middletemporal L |
|  | superiorparietal R |  | middletemporal R |
| **Cingulo-Opercular Network (CON)** | caudalanteriorcingulate L |  | parahippocampal L |
|  | caudalanteriorcingulate R |  | parahippocampal R |
|  | insula L |  | superiortemporal L |
|  | insula R |  | superiortemporal R |
|  | parsopercularis L |  | temporalpole L |
|  | parsopercularis R |  | temporalpole R |
|  | parsorbitalis L |  | bankssts L |
|  | parsorbitalis R | **Visual Network (Vis)** | cuneus L |
|  | parstriangularis L |  | cuneus R |
|  | parstriangularis R |  | fusiform L |
|  | superiorfrontal L |  | fusiform R |
|  | superiorfrontal R |  | lateraloccipital L |
|  | transversetemporal L |  | lateraloccipital R |
|  | transversetemporal R |  | lingual L |
| **anterior Default Mode Network (antDMN)** | frontalpole L |  | lingual R |
|  | frontalpole R |  | pericalcarine L |
|  | lateralorbitofrontal L |  | pericalcarine R |
|  | lateralorbitofrontal R | **Sensory Motor Network (SM)** | paracentral L |
|  | medialorbitofrontal L |  | paracentral R |
|  | medialorbitofrontal R |  | precentral L |
|  | rostralanteriorcingulate L |  | precentral R |
|  | rostralanteriorcingulate R |  | postcentral L |
| **posterior Default Mode Network (pDMN)** | inferiorparietal L |  | postcentral R |
|  | inferiorparietal R | **Ventral Attention Network (VAN)** | bankssts R |
|  | isthmuscingulate L |  | posteriorcingulate L |
|  | isthmuscingulate R |  | posteriorcingulate R |
|  | precuneus L |  | supramarginal L |
|  | precuneus R |  | supramarginal R |

**Table S1.** 8 brain networks and the cortical regions of interest (ROI) associated with each. The total 68 ROIs are from the Desikan-Killiany atlas [17]

| **Group Name** | **Electrode** | **Group Name** | **Electrode** |
| --- | --- | --- | --- |
| **Frontal Medial (FM)** | AFz | **Central** | Cz |
|  | Fz |  | CPz |
|  | FCz |  | C3 |
| **Posterior-Occipital Medial (POM)** | Pz |  | C4 |
|  | POz | **Frontal Left (FL)** | Fp1 |
|  | Oz |  | F3 |
| **Posterior-Occipital Left (POL)** | P3 |  | F7 |
|  | P7 |  | FC3 |
|  | O1 | **Frontal Right (FR)** | Fp2 |
| **Posterior Occipital Right (POR)** | P4 |  | F4 |
|  | P8 |  | F8 |
|  | O2 |  | FC4 |

**Table S2.** Scalp electrode groupings and the individual electrodes from the 24 channel EEG recording set-up associated with each electrode grouping.

| **Category** | **Dataset** | **Networks** | **Trial Events/Task** | **Behavior** | **# Variables** |
| --- | --- | --- | --- | --- | --- |
| **Primary: Task Related EEG Source Network Power** | IC | (8) Source networks (FPN, CON, aDMN, pDMN, mtl-DMN, Visual, SM, VAN) | (4) Trial Cue, Stimulus, Response, Feedback | None | 32 (8x4) |
|  | IP |  |  |  | 32 (8x4) |
|  | EB |  |  |  | 32 (8x4) |
|  | WM |  |  |  | 32 (8x4) |
|  | Rest |  | N/A |  | 8 |
| **Secondary: Task Related EEG Source Network Power + Task Performance** | IC | (8) Source networks (FPN, CON, aDMN, pDMN, mtl-DMN, Visual, SM, VAN) | (4) Trial Cue, Stimulus, Response, Feedback | (3) scaled d’ , RT, Consistency | 35 (8x4+3) |
|  | IP |  |  |  | 35 (8x4+3) |
|  | EB |  |  |  | 35 (8x4+3) |
|  | WM |  |  | (4) scaled d’ , RT, Consistency, WM item span | 36 (8x4+4) |
| **Secondary: Event Related EEG Source Network Power** | Cue | (8) Source networks (FPN, CON, aDMN, pDMN, mtl-DMN, Visual, SM, VAN) | (4) Tasks: IC, IP, EB, WM | None | 32 (8x4) |
|  | Stim |  |  |  | 32 (8x4) |
|  | Response |  |  |  | 32 (8x4) |
|  | Feedback |  |  |  | 32 (8x4) |
| **Secondary: Task Related EEG Scalp Power** | IC | (7) Electrode Groups (FM, POM, POL, POR, Central, FL, FR) | (4) Trial Cue, Stimulus, Response, Feedback | None | 28 (7x4) |
|  | IP |  |  |  | 28 (7x4) |
|  | EB |  |  |  | 28 (7x4) |
|  | WM |  |  |  | 28 (7x4) |

**Table S3:** Breakdown of the variables in each dataset in each category of ML models. All datasets are repeated 3 times for theta, alpha, and beta band power. IC: Inhibitory Control task, IP: Interference Processing task, WM: Working Memory task, EB: Emotion Bias task**.**

**Supplementary Figures**


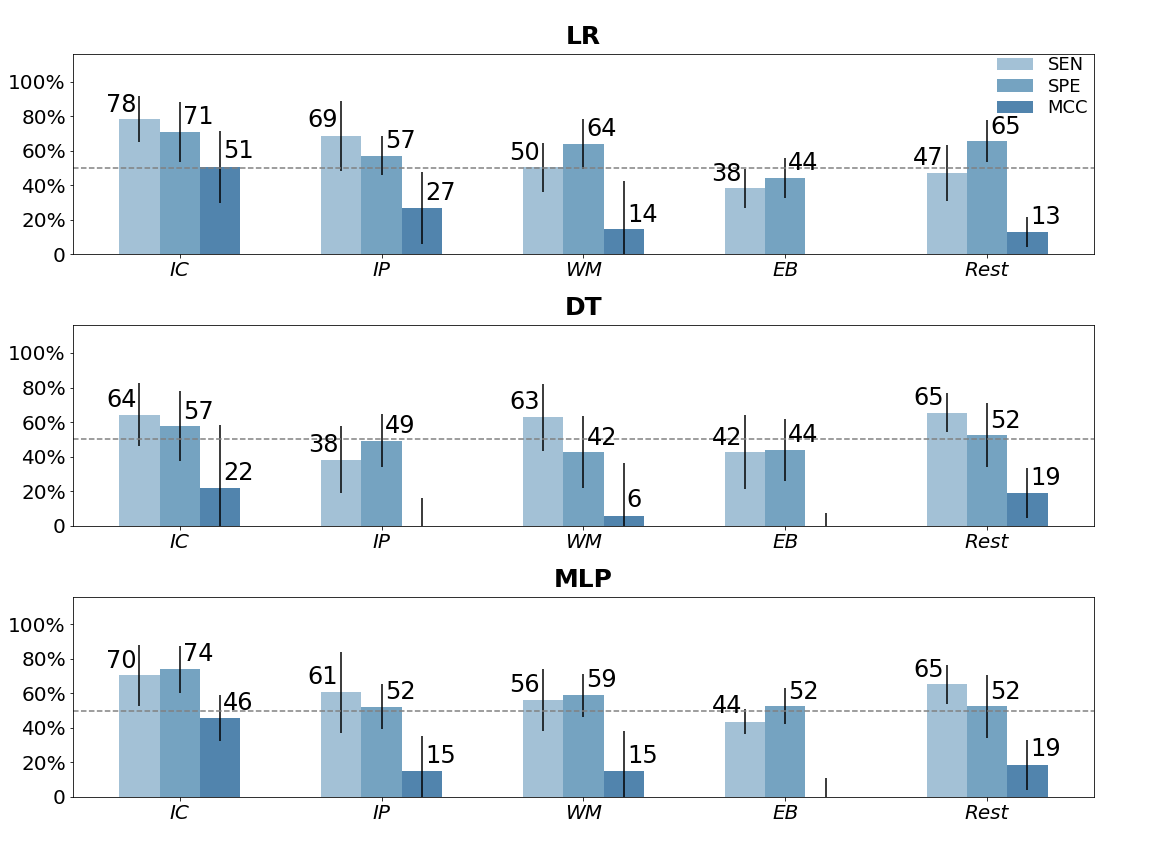


**Figure S1:** Theta band models of primary category for task related EEG source network power. Three rows correspond to LR: Logistic Regression, DT: Decision Tree and MLP: Multilayer Perceptron models.

**
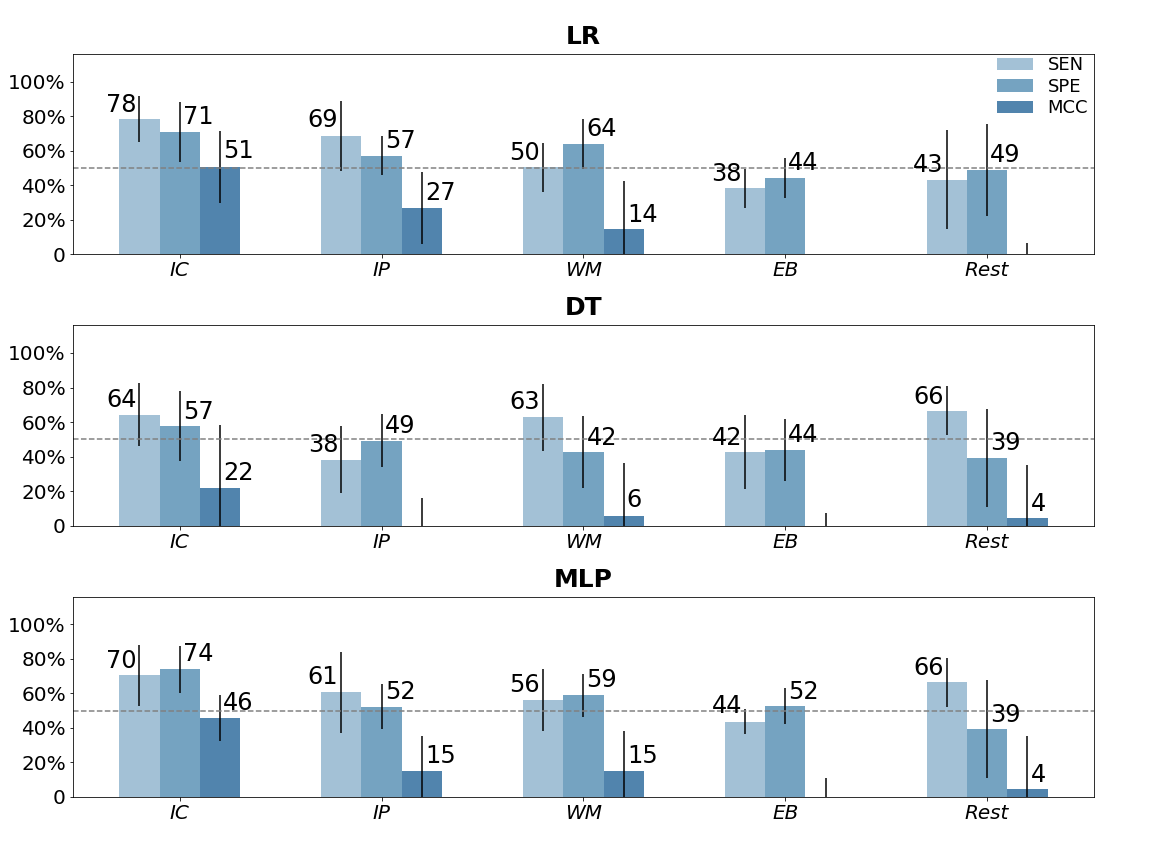
**

**Figure S2:** Alpha band model of primary category for task related EEG source network power. Three rows correspond to LR: Logistic Regression, DT: Decision Tree and MLP: Multilayer Perceptron models.

**References**

1. Greenberg LM, Waldmant ID. Developmental Normative Data on The Test of Variables of Attention (T.O.V.A.^TM^). Journal of Child Psychology and Psychiatry. 1993;34:1019–1030.

2. Fakhraei L, Francoeur M, Balasubramani P, Tang T, Hulyalkar S, Buscher N, et al. Mapping Large-Scale Networks Associated with Action, Behavioral Inhibition and Impulsivity. ENeuro. 2021;8:ENEURO.0406-20.2021.

3. Eriksen BA, Eriksen CW. Effects of noise letters upon identification of a target letter in a non-search task. Percept Psychophys. 1974;16.

4. Lavie N, Hirst A, Fockert JW, Viding E. Load theory of selective attention and cognitive control. J Exp Psychol Gen. 2004;133.

5. Shipstead Z, Harrison TL, Engle RW. Working Memory Capacity and Visual Attention: Top-Down and Bottom-Up Guidance. Quarterly Journal of Experimental Psychology. 2012;65:401–407.

6. Sternberg S. High-speed scanning in human memory. Science (80-). 1966;153.

7. Lenartowicz A. Electroencephalography correlates of spatial working memory deficits in attention-deficit/hyperactivity disorder: vigilance, encoding, and maintenance. J Neurosci. 2014;34.

8. López-Martín S, Albert J, Fernández-Jaén A, Carretié L. Emotional response inhibition in children with attention-deficit/hyperactivity disorder: neural and behavioural data. Psychol Med. 2015;45.

9. López-Martín S, Albert J, Fernández-Jaén A, Carretié L. Emotional distraction in boys with ADHD: Neural and behavioral correlates. Brain Cogn. 2013;83.

10. Thai N, Taber-Thomas BC, Pérez-Edgar KE. Neural correlates of attention biases, behavioral inhibition, and social anxiety in children: An ERP study. Dev Cogn Neurosci. 2016;19:200–210.

11. Tottenham N. The NimStim set of facial expressions: Judgments from untrained research participants. Psychiatry Res. 2009;168.

12. Heeger D, Landy M. Signal Detection Theory. Encyclopedia of Perception. 2009:887–892.

13. Delorme A, Makeig S. EEGLAB: an open source toolbox for analysis of single-trial EEG dynamics including independent component analysis. J Neurosci Methods. 2004;134.

14. Ojeda A, Kreutz-Delgado K, Mullen T. Fast and robust Block-Sparse Bayesian learning for EEG source imaging. Neuroimage. 2018;174.

15. Ojeda A, Kreutz-Delgado K, Mishra J. Bridging M/EEG Source Imaging and Independent Component Analysis Frameworks Using Biologically Inspired Sparsity Priors. Neural Comput. 2021;33:2408–2438.

16. Balasubramani PP, Ojeda A, Grennan G, Maric V, Le H, Alim F, et al. Mapping cognitive brain functions at scale. Neuroimage. 2021;231:117641.

17. Desikan RS, Ségonne F, Fischl B, Quinn BT, Dickerson BC, Blacker D, et al. An automated labeling system for subdividing the human cerebral cortex on MRI scans into gyral based regions of interest. Neuroimage. 2006;31:968–980.

18. Holmes CJ, Hoge R, Collins L, Woods R, Toga AW, Evans AC. Enhancement of MR Images Using Registration for Signal Averaging. J Comput Assist Tomogr. 1998;22:324–333.

19. Pascual-Marqui RD, Michel CM, Lehmann D. Low resolution electromagnetic tomography: a new method for localizing electrical activity in the brain. International Journal of Psychophysiology. 1994;18:49–65.

20. Ding L, He B. Sparse source imaging in electroencephalography with accurate field modeling. Hum Brain Mapp. 2008;29:1053–1067.

21. Stopczynski A, Stahlhut C, Larsen JE, Petersen MK, Hansen LK. The Smartphone Brain Scanner: A Portable Real-Time Neuroimaging System. PLoS One. 2014;9:e86733.

22. Shah R V., Grennan G, Zafar-Khan M, Alim F, Dey S, Ramanathan D, et al. Personalized machine learning of depressed mood using wearables. Transl Psychiatry. 2021;11:338.
